# Delta opioid peptide [d-Ala2, d-Leu5] enkephalin confers neuroprotection by activating delta opioid receptor-AMPK-autophagy axis against global ischemia

**DOI:** 10.1186/s13578-020-00441-z

**Published:** 2020-06-15

**Authors:** Zelin Lai, Lingling Gu, Lu Yu, Huifen Chen, Zhenhua Yu, Cheng Zhang, Xiaoqing Xu, Mutian Zhang, Min Zhang, Mingliang Ma, Zheng Zhao, Jun Zhang

**Affiliations:** 1 Key Laboratory of Brain Functional Genomics (East China Normal University), Ministry of Education, School of Life Sciences, East China Normal University, Shanghai, 200062 China; 2 Comprehensive Department of Traditional Chinese Medicine, Putuo Hospital, Affiliated to Shanghai University of Traditional Chinese Medicine, Shanghai, 200062 China; 3 Department of Clinical Laboratory, Shanghai First Maternity and Infant Hospital, Tongji University School of Medicine, Shanghai, 201204 China; 4 Department of Clinical Laboratory, Shanghai Public Health Clinical Center, Affiliated to Fudan University, Shanghai, 201508 China; 5 Shanghai Engineering Research Center of Molecular Therapeutics and New Drug Development, School of Chemistry and Molecular Engineering, East China Normal University, Shanghai, 200062 China

**Keywords:** Ischemia/reperfusion injury, Hippocampus, Delta opioid receptor, Neuronal autophagy, AMPK/mTOR/ULK1 signaling pathway

## Abstract

**Background:**

Ischemic stroke poses a severe risk to human health worldwide, and currently, clinical therapies for the disease are limited. Delta opioid receptor (DOR)-mediated neuroprotective effects against ischemia have attracted increasing attention in recent years. Our previous studies revealed that DOR activation by [d-Ala2, d-Leu5] enkephalin (DADLE), a selective DOR agonist, can promote hippocampal neuronal survival on day 3 after ischemia. However, the specific molecular and cellular mechanisms underlying the DOR-induced improvements in ischemic neuronal survival remain unclear.

**Results:**

We first detected the cytoprotective effects of DADLE in an oxygen–glucose deprivation/reperfusion (OGD/R) model and observed increased viability of OGD/R SH-SY5Y neuronal cells. We also evaluated changes in the DOR level following ischemia/reperfusion (I/R) injury and DADLE treatment and found that DADLE increased DOR levels after ischemia in vivo and vitro. The effects of DOR activation on postischemic autophagy were then investigated, and the results of the animal experiment showed that DOR activation by DADLE enhanced autophagy after ischemia, as indicated by elevated LC3 II/I levels and reduced P62 levels. Furthermore, the DOR-mediated protective effects on ischemic CA1 neurons were abolished by the autophagy inhibitor 3-methyladenine (3-MA). Moreover, the results of the cell experiments revealed that DOR activation not only augmented autophagy after OGD/R injury but also alleviated autophagic flux dysfunction. The molecular pathway underlying DOR-mediated autophagy under ischemic conditions was subsequently studied, and the in vivo and vitro data showed that DOR activation elevated autophagy postischemia by triggering the AMPK/mTOR/ULK1 signaling pathway, while the addition of the AMPK inhibitor compound C eliminated the protective effects of DOR against I/R injury.

**Conclusion:**

DADLE-evoked DOR activation enhanced neuronal autophagy through activating the AMPK/mTOR/ULK1 signaling pathway to improve neuronal survival and exert neuroprotective effects against ischemia.

## Background

Stroke is an acute cerebrovascular disease with high disability and mortality. According to a systematic country-specific analysis on the lifetime risk of stroke, China has the highest estimated risk worldwide [1]. Meanwhile, stroke has been the leading cause of death in China [2]. The lifetime risk of ischemic stroke far exceeds that of hemorrhagic stroke [1]. However, there are currently obvious limitations (e.g., a narrow therapeutic window and selective efficacy) on clinical therapies for ischemic stroke, partly due to the limited understanding of disease pathogenesis. Therefore, further studies on the pathological mechanisms of the disease and potential therapeutic strategies are necessary.

Delta opioid receptor (DOR) is a G protein-coupled receptor (GPCR) that is highly expressed in the hippocampus, striatum and other brain regions involved in learning and memory [3]. Growing evidence has revealed the beneficial biochemical and physiological effects of DOR in depression/anxiety, addiction, and parkinsonian injury [3–5]. In recent years, interest has increasingly shifted to the neuroprotective effects of DOR against cerebral ischemia. For instance, DOR can promote neurological recovery in different ischemic models (e.g., the middle cerebral artery occlusion (MCAO) model and the four-vessel occlusion (4-VO) model) after treatment with the nonpeptidic DOR agonist Tan-67 [6] or the delta opioid peptide [D-Ala2, D-Leu5] enkephalin (DADLE) [7]. Mechanistic studies have further shown that DOR signaling triggers different neuroprotective mechanisms, such as ionic homeostasis maintenance [8], glutamate excitotoxicity alleviation [9], and Bax-related apoptosis inhibition [10, 11], in response to ischemic stress. Multiple molecular signaling pathways, including the PKC/ERK [12], PI3K/Akt [7, 13], and BDNF/TrkB [14] pathways, have been demonstrated to be involved in DOR-mediated neuroprotection, suggesting that DOR could be a potential target against cerebral ischemia and neurological impairment.

Autophagy is a cellular catabolic process that confers cytoprotection against various pathological stresses, including ischemia/reperfusion (I/R) injury [15–17]. In vivo imaging of focal ischemic mice revealed that GFP-LC3 fluorescence can be observed in neurons in the ischemic hemisphere [18]. Autophagic activation is also evident in ischemic astrocytes [19]. Previous data have indicated that autophagy is strongly associated with AMPK [20] and that pharmacological inhibition of AMPK-modulated autophagy can neutralize the anti-ischemic effects of ischemic preconditioning [21] or metformin [22], implying that AMPK-mediated autophagy plays an important role in neuroprotection against cerebral ischemia.

DADLE is a synthetic DOR agonist, and in vitro and in vivo studies have revealed that DADLE-evoked DOR activation attenuates neuronal damage induced by I/R injury [10, 23]. Our previous studies also demonstrated that DADLE treatment not only significantly improves neuronal survival and astrocytic activation but also ameliorates cognitive impairment in global ischemia rats [7, 13]. Furthermore, in an in vitro study, we observed that DADLE induces autophagy in astrocytes in response to oxygen-glucose deprivation (OGD) injury [11], providing preliminary evidence of an interaction between DOR-mediated autophagy and neuronal protection in cerebral ischemic injury. Despite these findings, however, the exact molecular and cellular mechanisms responsible for the anti-ischemic actions of DOR-induced autophagy remain unclear. This study was therefore conducted to first examine the expression changes in DOR in response to I/R injury as well as DADLE treatment and then explore whether such responses could lead to autophagy activation through modulation of AMPK signaling to eventually determine neuronal fate both in vivo in the hippocampi of global ischemia rats and in vitro in an oxygen-glucose deprivation/reperfusion (OGD/R) cell model.

## Methods

### Animals

All experiments involving animals were performed in accordance with the Animals Act (2006, China) and approved by the Institutional Animal Care and Use Committee of East China Normal University (IACUC approval ID AR201404022). Male Sprague–Dawley rats (mean body weight of 200–250 g) were purchased from Shanghai Laboratory Animal Center (Shanghai, China). The rats were individually housed and maintained on a 12-h light/dark cycle under constant temperature and humidity. The animals had free access to water and food. Efforts were made to minimize the number of animals used and their suffering.

### Intracerebral cannulation and global cerebral ischemia model

**Fig. 1:**
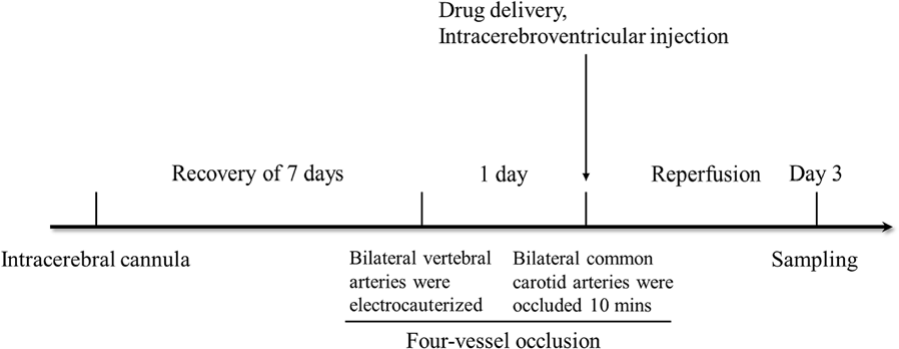
Diagram of the animal experimental procedure, including intracerebral cannula implantation, four-vessel occlusion and intracerebroventricular injection

As shown in Fig. 1, rats were anesthetized with 10% chloral hydrate (300 mg/kg, i.p.), and a cannula was implanted into the right lateral ventricle. The animals were placed in a stereotaxic frame with the skull positioned horizontally. A guide cannula was positioned at the following coordinates: 0.8 mm posterior to bregma, 1.5 mm lateral to the midline and 3.8 mm ventral to the skull surface. All coordinates were derived from the atlas of Paxinos and Watson [24]. The guide cannula and 2 stainless steel screws were anchored to the skull with acrylic dental cement. The rats underwent cerebral ischemia surgery after at least 6 days of recovery.

Global cerebral ischemia induced by 4-VO as described previously [7, 13]. In brief, the rats were anesthetized with 10% chloral hydrate (300 mg/kg, i.p.), and the bilateral vertebral arteries were electrocauterized with an electrocoagulator through the alar foramina of the first cervical vertebra. Both common carotid arteries were exposed through a ventral midline cervical incision, and small-diameter ligatures were placed loosely around each artery without interrupting the carotid blood flow to facilitate subsequent occlusion. The animals were allowed to recover from anesthesia and were fasted before the next operation. On the following day, the rats were light anesthetized with ethyl ether, and the bilateral common carotid arteries were re-exposed. After the rats awoke, the bilateral common carotid arteries were occluded for 10 min with artery clips. Ischemia was confirmed by monitoring the loss of righting reflex and bilateral pupil dilation during carotid occlusion. Sham-operated rats underwent the same surgical procedures except for carotid artery occlusion. Core body temperature was monitored with a rectal probe and maintained at 37 °C throughout the experiment.

### Groups and drug administration

As shown in Table 1, for immunoblotting of relevant proteins, the animals were assigned to 4 groups (N = 3 per group): the sham group, I/R group, DADLE group and DADLE + naltrindole group. To study the effects of autophagy in ischemic neurons following DADLE treatment, the animals were assigned to 5 groups (N = 3 per group): the sham group, I/R group, DADLE group, DADLE + naltrindole group and DADLE + 3-methyladenine (3-MA) group. To evaluate the role of the AMPK cascade in DADLE-mediated neuroprotection, the animals were assigned to 6 groups (N = 3 per group): the sham group, I/R group, DADLE group, DADLE + compound C group, A769662 group and compound C group.

**Table 1:** Groups and treatments used in the animal experiments

Experimental groups	Experimental treatments
Sham	Intracerebral cannula + electrocoagulation of the vertebral arteries + PBS (total 7.5 μl)
I/R	Intracerebral cannula + electrocoagulation of the vertebral arteries + carotid artery occlusion + PBS (total 7.5 μl)
DADLE	Intracerebral cannula + electrocoagulation of the vertebral arteries + carotid artery occlusion + 2.5 nmol DADLE (total 7.5 μl)
DADLE + naltrindole	Intracerebral cannula + electrocoagulation of the vertebral arteries + carotid artery occlusion + 2.5 nmol DADLE + 2.5 nmol naltrindole (total 7.5 μl)
DADLE + 3-MA	Intracerebral cannula + electrocoagulation of the vertebral arteries + carotid artery occlusion + 2.5 nmol DADLE + 600 nmol 3-MA (total 7.5 μl)
DADLE + compound C	Intracerebral cannula + electrocoagulation of the vertebral arteries + carotid artery occlusion + 2.5 nmol DADLE + 100 nmol compound C (total 7.5 μl)
A769662	Intracerebral cannula + electrocoagulation of the vertebral arteries + carotid artery occlusion + 100 nmol A769662 (total 7.5 μl)
Compound C	Intracerebral cannula + electrocoagulation of the vertebral arteries + carotid artery occlusion + 100 nmol compound C (total 7.5 μl)

For drug delivery of the DOR agonist and antagonist, 7.5 μl phosphate buffer saline (PBS), PBS containing 2.5 nmol DADLE (Tocris, UK) or PBS containing 2.5 nmol DADLE and 2.5 nmol naltrindole (Tocris) was administered slowly (approximately 2 min) at the onset of reperfusion. PBS (7.5 μl) containing 2.5 nmol DADLE and 600 nmol 3-MA (Selleck, USA), as an autophagy inhibitor, was administered. For drug delivery of the AMPK agonist and antagonist, 7.5 μl PBS containing 100 nmol A769662 (Selleck), PBS containing 100 nmol compound C (Selleck) or PBS containing 2.5 nmol DADLE and 100 nmol compound C was administered. To minimize leakage of fluid from the injection site, the needle was left in place for 30 s before retraction.

### Perfusion and tissue preparation

Rats were anesthetized with 10% chloral hydrate on day 3 postischemia and perfused transcardially with normal saline followed by 4% ice-cold paraformaldehyde (PFA). The brains were then removed immediately submerged in 4% PFA solution overnight at 4 °C, and dehydrated in gradient sucrose. Coronal sections (30 μm) of brain tissue containing the hippocampus were stored at − 80 °C.

### Immunofluorescence assay

The brain sections were heated in antigen retrieval solution at 95 °C for 10 min. After they were cooled, the sections were blocked with 5% goat serum for 2 h at room temperature. Then they were incubated with primary antibody against neuron-specific nuclear protein (NeuN) (1:500, Abcam, UK) overnight at 4 °C followed by Cy3-conjugated secondary antibody for 2 h at room temperature. The brain sections were observed with a fluorescence microscope (Leica, Germany).

### Western blotting

Hippocampal proteins and cellular proteins were extracted in RIPA lysis buffer containing 1 mM PMSF (Beyotime, China). The proteins were separated by 8–12% SDS-PAGE and electrotransferred onto PVDF membranes. After being blocked with 5% skim milk, the membranes were incubated with primary antibodies against DOR, LC3 (1:1000, Abcam), P62, β-actin, AMPKα, p-AMPKα T172, mTOR, p-mTOR S2448, ULK1, p-ULK1 S317, and p-ULK1 S757 (1:1000, Cell Signaling Technology, USA) overnight at 4 °C. Afterwards, they were incubated with secondary antibodies for 2 h at room temperature and then scanned with a Bio-Rad gel imaging system.

### OGD/R model and groups

Human neuroblastoma SH-SY5Y cells were obtained from the Cell Bank of the Chinese Academy of Sciences. The cells were cultured in DMEM/F12 with 10% fetal bovine serum (Gibco, USA), 50 μg/ml streptomycin and 50 IU/ml penicillin and maintained at 37 °C in a humidified atmosphere containing 5% CO_2_.

SH-SY5Y cells were washed twice with PBS, and the medium was replaced with glucose-free balanced salt solution containing 135 mM NaCl, 4.7 mM KCl, 10 mM Na_2_HPO_4_, and 2 mM NaH_2_PO_4_, pH 7.4. They were then incubated in a hypoxia chamber filled with 95% N_2_/5% CO_2_ for 2 h. After OGD, the cells were cultured again in complete culture medium under normoxic conditions for 0.5 h. As shown in Table 2, 20 μM DADLE, 20 μM naltrindole, 5 mM 3-MA, 100 μM compound C or a combination of these drugs was added to the different experimental groups at the beginning of OGD.

**Table 2:** Groups and treatments used in the cell experiments

Experimental groups	Experimental treatments
Control	Normal culture
OGD/R	OGD/R + PBS
DADLE	OGD/R + 20 μM DADLE
Naltrindole	OGD/R + 20 μM naltrindole
DADLE + naltrindole	OGD/R + 20 μM DADLE + 20 μM naltrindole
DADLE + 3-MA	OGD/R + 20 μM DADLE + 5 mM 3-MA
DADLE + compound C	OGD/R + 20 μM DADLE + 100 μM compound C

### Cell viability assay

Cell viability was examined using the Cell Counting Kit-8 (CCK-8). SH-SY5Y cells were cultured in a 96-well plate and underwent experimental treatment. Then, CCK-8 reagent was subsequently added to each well. The cells were incubated at 37 °C for 1–2 h. The optical density (OD) was measured at 450 nm with a Spectra Max M5 reader (Molecular Devices, USA).

### Autophagic flux assay

Cells were grown on 24-well plates and grown to 30% confluence before transfection. They were transfected with Ad-mCherry-GFP-LC3B at a multiplicity of infection (MOI) of 20 and exposed to the experimental conditions as described above. Autophagic flux was observed by a laser scanning confocal microscope (Leica) and evaluated by calculating the number of red and yellow dots.

### Monodansylcadaverine (MDC) staining

Autophagy was measured using MDC staining. Treated cells were washed twice with wash buffer and stained with MDC staining solution for 40 min at room temperature. They were washed 3 times with wash buffer, fixed with 4% PFA at room temperature, and washed 3 times with wash buffer. The cells were observed with a laser scanning confocal microscope (Leica) and evaluated by calculating the number of green dots.

### Data analysis

ImageJ was used for image processing. Statistical analysis was performed with GraphPad Prism 7. The values are expressed as the mean ± SEM. Student’s t-test was used to compare two groups. Multiple data sets were analyzed using one-way analysis of variance followed by Tukey’s multiple comparisons test. Differences were considered significant at P < 0.05, as indicated by *.

## Results

### DOR expression is changed in response to I/R injury and DADLE treatment

**Fig. 2:**
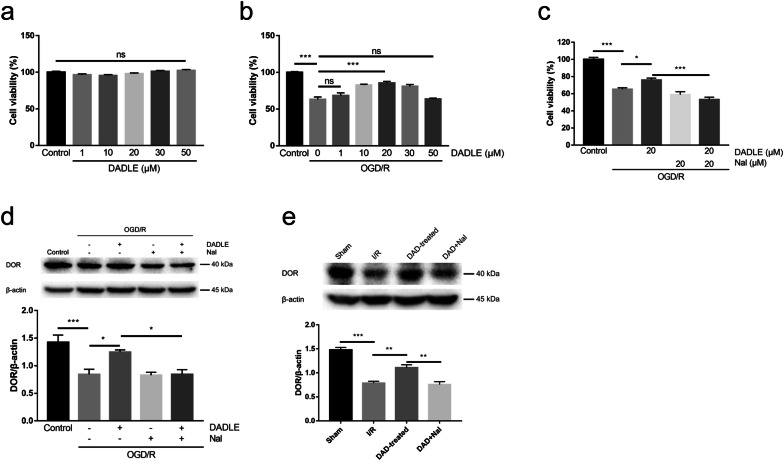
DADLE exerted cytoprotective effects in OGD/R cells and elevated postischemic DOR levels in vivo and vitro. The viability of SH-SY5Y cells exposed to gradient concentrations of DADLE in normal culture (**a**) and under OGD/R conditions (**b**) (N = 4 per group, three independent experiments). **c** The viability of SH-SY5Y cells treated with OGD/R and DADLE, naltrindole or DADLE + naltrindole (N = 4 per group, three independent experiments). **d** Western blot analysis of DOR expression in SH-SY5Y cells following OGD/R injury (N = 6 per group). **e** Western blot analysis of DOR levels in the rat hippocampus on day 3 after ischemia (N = 3 per group). The values are shown as the mean ± SEM. There were no significant differences between groups in **a**. Significant differences: the OGD/R group vs. the 10 μM, 20 μM and 30 μM DADLE groups (***p < 0.001) in **b**; the OGD/R group vs. the DADLE group (*p < 0.05) and the naltrindole group (p > 0.05, ns), the DADLE group vs. the DADLE + naltrindole group (***p < 0.001) in **c**; the OGD/R group vs. the DADLE group (*p < 0.05) and the naltrindole group (p > 0.05, ns), the DADLE group vs. the DADLE + naltrindole group (*p < 0.05) in **d**; the I/R group vs. the DADLE group (**p < 0.01) and the DADLE group vs. the DADLE + naltrindole group (**p < 0.01) in **e**. *DAD* DADLE, *Nal* naltrindole

As we previously showed that DADLE treatment exerts neuroprotective effects in vivo in ischemic rats [7], we first assessed the role of DADLE in vitro in SH-SY5Y cells exposed to OGD/R injury in this study. The cell toxicity assays showed that the viability of SH-SY5Y cells treated with 1–50 μM DADLE was unchanged (Fig. 2a) but that the viability of OGD/R-exposed cells treated with 10–30 μM DADLE was markedly increased, with peak viability being observed at a dose of 20 μM (Fig. 2b). Moreover, the treatment of the DOR inhibitor naltrindole alone had no obvious changes in the viability of OGD/R-exposed cells, while this beneficial effect of DADLE was abolished in OGD/R-exposed cells treated with naltrindole in combination with DADLE (Fig. 2c), implying that DADLE exerts DOR-dependent cytoprotective effects against OGD/R injury. We then examined the changes in DOR levels in OGD/R-exposed cells treated with or without DADLE. DOR expression was significantly downregulated in response to OGD/R insult, and this downregulation was obviously rescued by DADLE treatment and was unchanged by naltrindole treatment (Fig. 2d). Moreover, the DADLE-mediated upregulation of DOR in OGD/R cells was neutralized by DADLE + naltrindole (Fig. 2d). These cellular observations were confirmed in vivo in the hippocampi of ischemic rats; the level of hippocampal DOR was significantly increased in the DADLE group compared to the I/R group, and the DOR-stimulating effects of DADLE were counteracted by naltrindole (Fig. 2e). Together, the above data suggest that cerebral ischemia impairs the function of DOR and that DADLE treatment can at least partially restore the DOR level after I/R injury in vivo and in vitro.

### DOR activation augments autophagy to promote ischemic CA1 neuronal survival

**Fig. 3:**
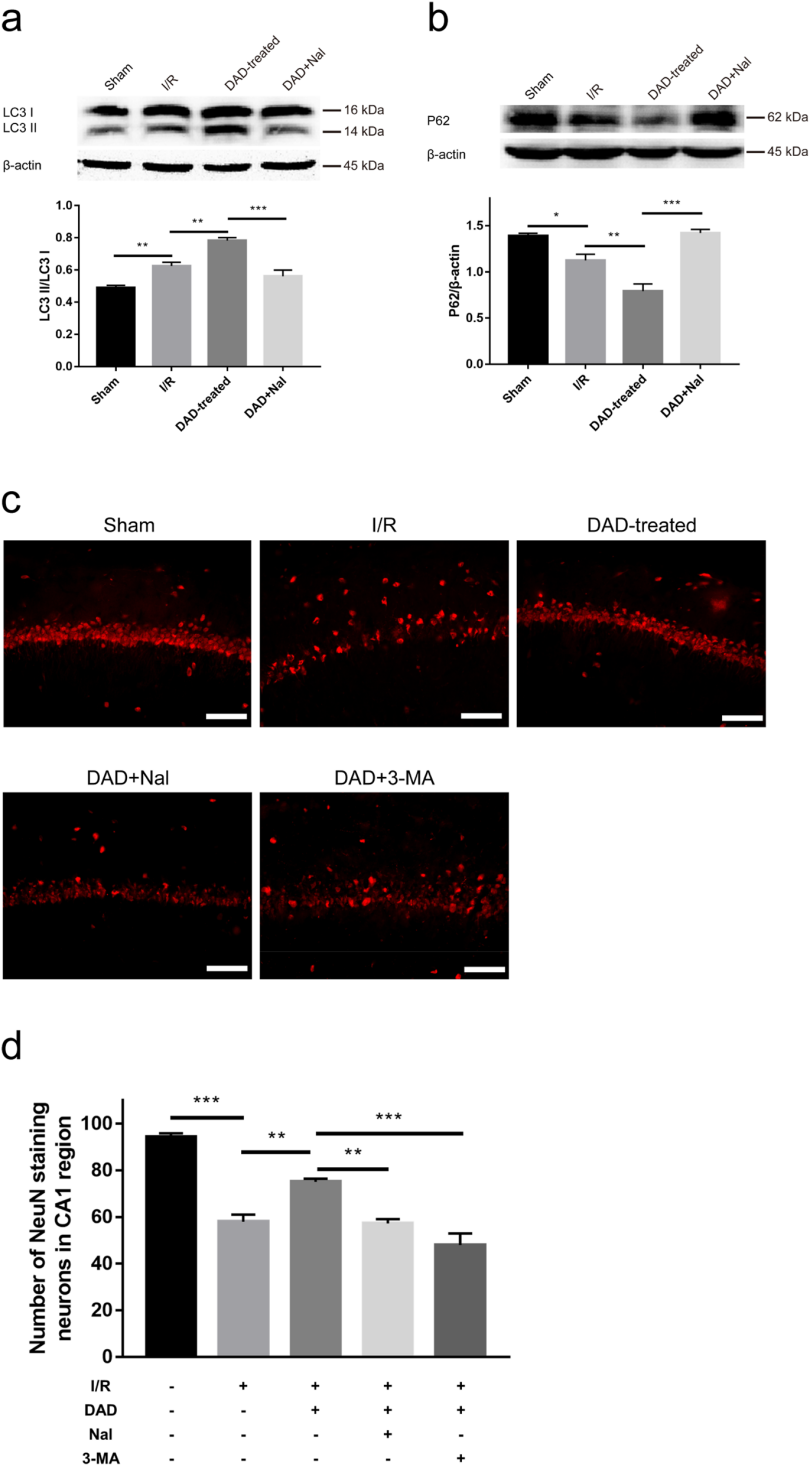
The effects of DOR-mediated autophagy on neuronal survival in the rat hippocampus on day 3 postischemia. Western blot analysis of LC3 II/I (**a**) and P62 (**b**) levels in the hippocampus (N = 3 per group). **c** Immunofluorescence images of NeuN-positive neurons in the CA1 region. Obvious neuronal loss was observed in the I/R group, DADLE + naltrindole group, and DADLE + 3-MA group compared with the sham group, while there was an obvious amelioration of neuronal survival in the DADLE group compared to the I/R group. Scale bar = 100 μm. **d** Quantitative analysis of neuron counts (N = 3 per group). The values are shown as the mean ± SEM. Significant differences: the I/R group vs. the DADLE group (**p < 0.01) and the DADLE group vs. the DADLE + naltrindole group (***p < 0.001) in **a**; the I/R group and vs. the DADLE group (**p < 0.01) and the DADLE group vs. the DADLE + naltrindole group (***p < 0.001) in **b**; the I/R group vs. the DADLE group (**p < 0.01) and the DADLE group vs. the DADLE + naltrindole group (**p < 0.01) and the DADLE + 3-MA group (***p < 0.001) in **d**. *DAD* DADLE, *Nal* naltrindole

Given that DADLE-induced DOR activation protects OGD-injured astrocytes by inducing autophagy in vitro [11], we examined the changes in autophagy in vivo in the rat hippocampus following ischemic injury and DADLE treatment. Elevated LC3 II/I levels and reduced P62 levels, indicating an endogenous autophagic response to ischemic stress, were observed in ischemic rats (Fig. 3a, b). The level of LC3 II/I was further elevated, and P62 was further reduced in the DADLE group compared to the I/R group, while the directions of these changes were reversed in the DADLE + naltrindole group (Fig. 3a, b), implying that DOR activation promoted postischemic autophagy. To determine whether DADLE-mediated autophagy is responsible for neuronal protection against I/R injury, survival of NeuN-labeled hippocampal neurons in the rats was observed on day 3 postischemia. The results showed obviously fewer CA1 neurons in the I/R group than the sham group and a significant improvement in neuronal survival in the DADLE group compared to the I/R group (Fig. 3c, d). The beneficial effects of DADLE were abolished by naltrindole or the autophagy inhibitor 3-MA (Fig. 3c, d). There was no significant neuronal injury in the CA3 region or the dentate gyrus (DG) (Additional files 1, 2: Figs. S1, S2), suggesting that there are differences in the ischemic tolerance of CA1, CA3 and DG neurons. These results suggest that DOR activation-induced autophagy can improve ischemic neuronal survival in the hippocampal CA1 region.

### DOR activation ameliorates autophagic flux dysfunction in OGD/R-exposed cells

**Fig. 4:**
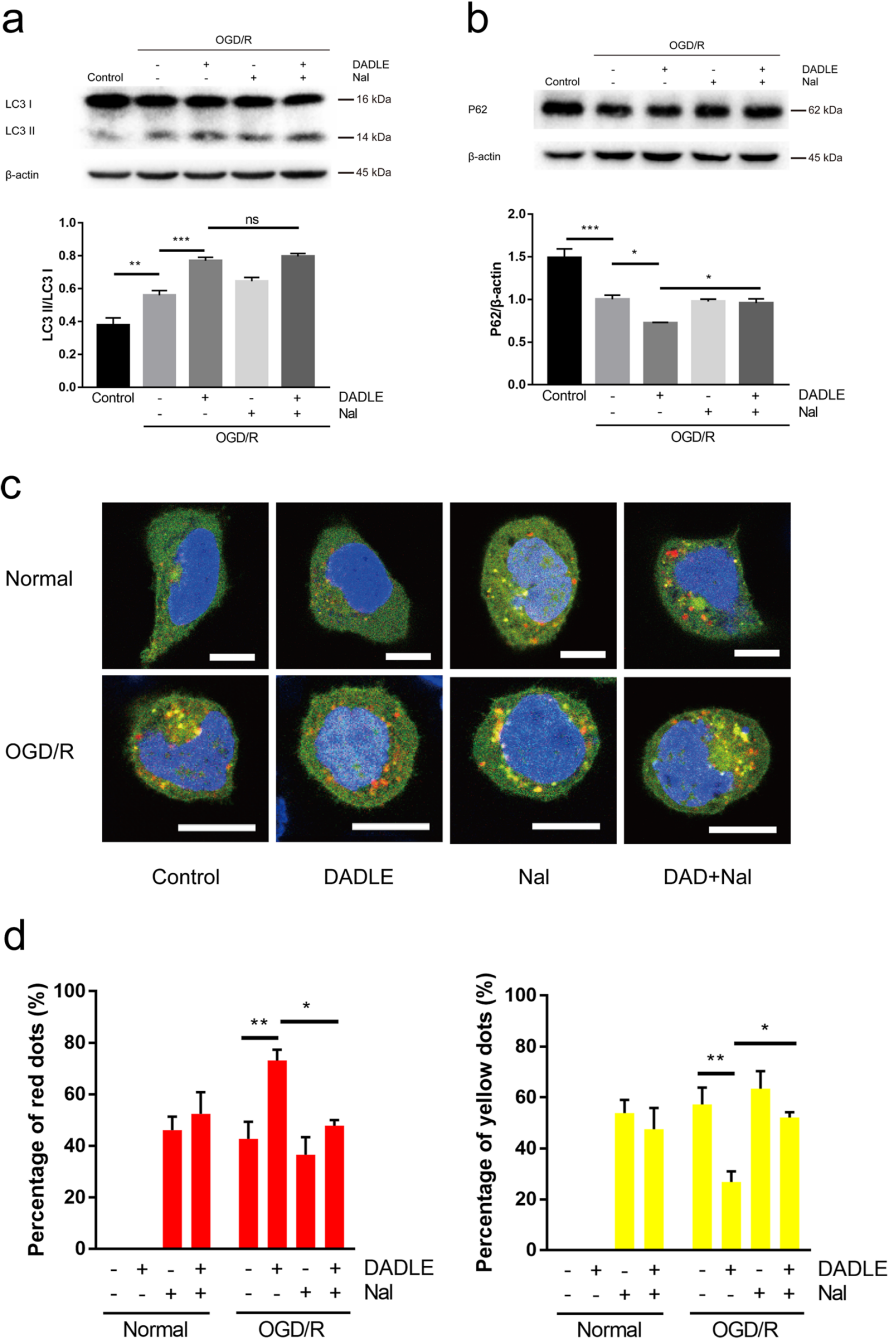
The effects of DOR activation on autophagy in SH-SY5Y cells after OGD/R injury. Western blot analysis of LC3 II/I (**a**) and P62 (**b**) levels in the OGD/R model (N = 6 per group). **c** Representative images of SH-SY5Y cells infected with Ad-mCherry-GFP-LC3B in each group. The yellow dots represent autophagosomes, and the red dots represent autolysosomes. Scale bar = 10 μm. **d** Statistical analysis of the percentages of red and yellow dots in cells (N = 5 per group). The values are shown as the mean ± SEM. Significant differences: the OGD/R group vs. the DADLE group (***p < 0.001) and the naltrindole group (p > 0.05, ns), the DADLE group vs. the DADLE + naltrindole group (p > 0.05, ns) in **a**; the OGD/R group vs. the DADLE group (*p < 0.05) and the naltrindole group (p > 0.05, ns), the DADLE group vs. the DADLE + naltrindole group (*p < 0.05) in **b**; the normal naltrindole group vs. the normal DADLE + naltrindole group (p > 0.05, ns), the OGD/R group vs. the DADLE group (**p < 0.01) and the naltrindole group (p > 0.05, ns), the DADLE group vs. the DADLE + naltrindole group (*p < 0.05) in **d**. *DAD* DADLE, *Nal* naltrindole

In parallel, we detected the effects of DOR on autophagy in SH-SY5Y cells after OGD/R- injury. The results showed that OGD/R injury elevated LC3 II/I levels and reduced P62 levels (Fig. 4a, b), suggesting that OGD/R injury activated an endogenous autophagic response in the cells. Compared with that in the OGD/R group, the LC3 II/I level was further increased, and the P62 level was further decreased in the DADLE group (Fig. 4a, b), indicating that DOR activation augmented autophagy after OGD/R injury. Notably, although the levels of LC3 II/I and P62 in the naltrindole group were similar to those in the OGD/R group (Fig. 4a, b), the level of LC3 II/I in the DADLE + naltrindole group unexpectedly appeared to be almost identical to that in the DADLE group (Fig. 4a) and to be inconsistent with the P62 level (Fig. 4b). Given that LC3 and P62 may have their own specific roles in different stages throughout the autophagy process, the observed inconsistent changes in LC3 and P62 might indicate that autophagic flux was abnormal in the OGD/R-exposed cells. Indeed, the expression of the mCherry-GFP-LC3B protein in adenovirus-infected cells revealed that autophagy was not activated in normal SH-SY5Y cells, as indicated by a lack of fluorescent dots (Fig. 4c). Similar fluorescence signal was also observed in the DADLE group, while red and yellow dots appeared in the naltrindole group and DADLE + naltrindole group (Fig. 4c). The proportions of red and yellow dots were calculated to be 46.15% and 53.85%, respectively, in the naltrindole group and 52.49% and 47.51%, respectively, in the DADLE + naltrindole group (Fig. 4d), indicating that autophagy occurred in the cells. After OGD/R, however, many yellow dots and a small number of red dots were observed in the OGD/R group, naltrindole group and DADLE + naltrindole group, while many red dots and a small number of yellow dots were observed in the DADLE group (Fig. 4c). The proportions of red and yellow dots were calculated to be 42.74% and 57.26%, respectively, in the OGD/R group; 73.14% and 26.86%, respectively, in the DADLE group; 36.53% and 63.47%, respectively, in the naltrindole group; and 47.92% and 52.08%, respectively, in the DADLE + naltrindole group (Fig. 4d). These data suggest that although OGD/R injury induced autophagy, a substantial amount of undegraded autophagosomes (indicated by yellow dots) accumulated, which led to autophagic flux dysfunction in the cells. However, DADLE-mediated DOR activation enhanced autophagy and accelerated the degradation of autophagosomes (indicated by red dots) to alleviate autophagic flux dysfunction, possibly eventually alleviating OGD/R injury.

### DOR activation enhances postischemic autophagy through the AMPK/mTOR/ULK1 pathway

**Fig. 5:**
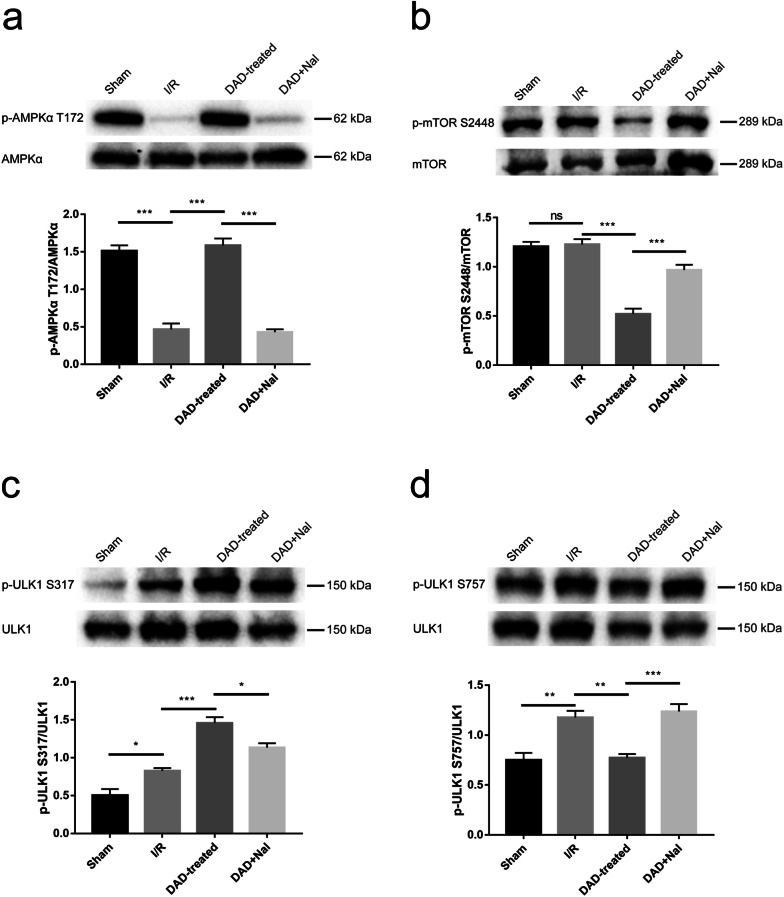
DOR activation triggered the AMPK/mTOR/ULK1 pathway to enhance postischemic autophagy. Western blot analysis of the phosphorylation levels of AMPKα T172 (**a**), mTOR S2448 (**b**), ULK1 S317 (**c**) and ULK1 S757 (**d**) in the rat hippocampus on day 3 after ischemia (N = 3 per group). The values are shown as the mean ± SEM. Significant differences: the I/R group vs. the DADLE group (***p < 0.001) and the DADLE group vs. the DADLE + naltrindole group (***p < 0.001) in **a**; the sham group vs. the I/R group (p > 0.05, ns), the I/R group vs. the DADLE group (***p < 0.001), and the DADLE group vs. the DADLE + naltrindole group (***p < 0.001) in **b**; the I/R group vs. the DADLE group (***p < 0.001) and the DADLE group vs. the DADLE + naltrindole group (*p < 0.05) in **c**; the I/R group vs. the DADLE group (**p < 0.01) and the DADLE group vs. the DADLE + naltrindole group (***p < 0.001) in **d**. *DAD* DADLE, *Nal* naltrindole

To reveal the molecular mechanisms that contribute to the DOR-mediated autophagy described above, the phosphorylation levels of proteins associated with the hippocampal AMPK/mTOR/ULK1 pathway were examined in rats 3 days after I/R injury with or without DADLE treatment. The phosphorylation of AMPKα T172 promotes autophagy by directly phosphorylating ULK1 S317, while p-mTOR S2448 prevents autophagic activation by phosphorylating ULK1 S757 [25]. The results showed that the phosphorylation level of AMPKα T172 was obviously decreased in the I/R rats compared to the sham rats, while it returned to normal levels upon DADLE treatment. This return to baseline was abolished by naltrindole (Fig. 5a). There was no significant change in the phosphorylation level of mTOR S2448 between the sham group and the I/R group. However, the phosphorylation level of mTOR S2448 was significantly decreased in the DADLE group compared to the I/R group, and this change was counteracted in the DADLE + naltrindole group (Fig. 5b). The p-ULK1 S317 level was increased in the I/R group compared with the sham group and was further increased in the DADLE group, while the increase in p-ULK1 S317 was suppressed in the DADLE + naltrindole group (Fig. 5c). In contrast with the p-ULK1 S317 level, the p-ULK1 S757 level was elevated in the I/R group and the DADLE + naltrindole group compared with the sham group and remained unchanged in the DADLE group (Fig. 5d). Together, these results suggest that the modulating effects of AMPK/mTOR/ULK1 signaling on endogenous autophagy were weakened on day 3 after ischemia, whereas DADLE-mediated DOR activation augmented autophagy by triggering AMPK/mTOR/ULK1 signaling.

**Fig. 6:**
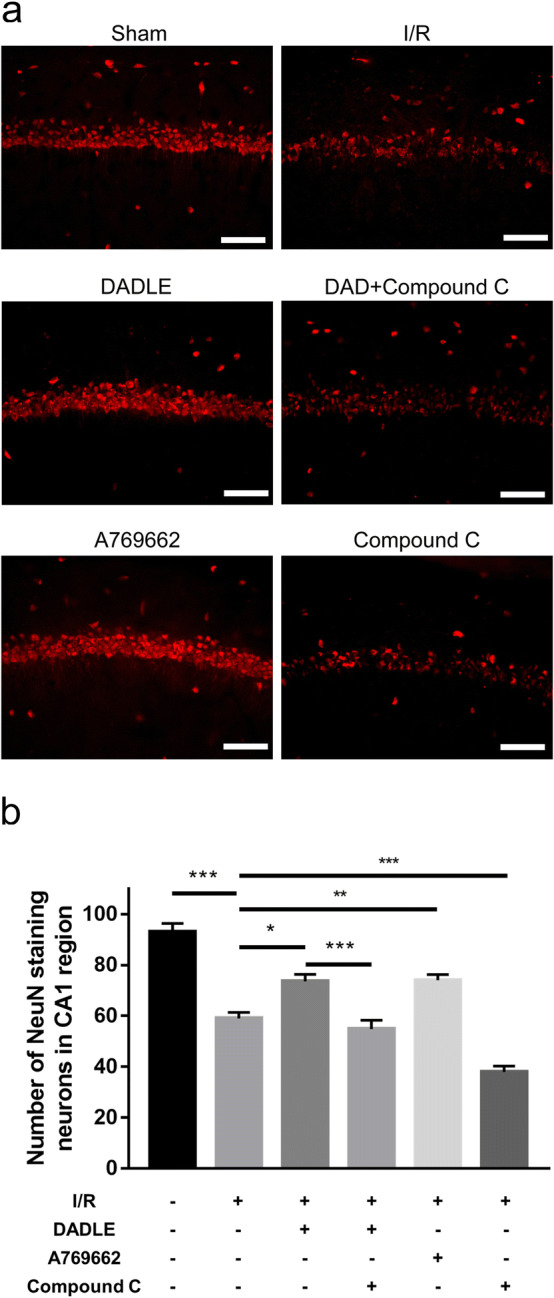
DOR-induced neuroprotection was AMPK-dependent in ischemic CA1 neurons. **a** Representative images of neurons labeled with NeuN. Obvious improvement in neuronal survival was observed in the DADLE group and A769662 group compared with the I/R group, while severe neuronal injury was observed in the compound C group. Obvious neuronal loss was observed in the DADLE + compound C group compared to the DADLE group. Scale bar = 100 μm. **b** Quantitative analysis of neuron counts (N = 3 per group). The values are shown as the mean ± SEM. Significant differences: the I/R group vs. the DADLE group (*p < 0.05), the A769662 group (**p < 0.01) and the compound C group (***p < 0.001), the DADLE group vs. the DADLE + compound C group (***p < 0.001) and the A769662 group (p > 0.05, ns) in **b**. DAD: DADLE

To further explore the role of AMPK in DOR-mediated neuroprotection, the effects of the AMPK agonist A769662 and inhibitor compound C in ischemic neurons were determined. CA1 neuronal survival in the sham group, I/R group and DADLE group was consistent with the results mentioned above (Figs. 3, 6), while the neuroprotective effects of DADLE in ischemic neurons were abolished by the addition of compound C (Fig. 6a, b). Similar to the neuroprotective effect induced by DADLE, AMPK activation with A769662 significantly improved the survival of ischemic neurons, while AMPK inhibition by compound C further aggravated neuronal damage (Fig. 6a, b). These in vivo results indicate that the effect of DADLE against ischemia could be attributed to a DOR/AMPK/mTOR/ULK1 signaling pathway-dependent increase in autophagic activity.

### DOR activation promotes autophagy in OGD/R-exposed cells in an AMPK-dependent manner

**Fig. 7:**
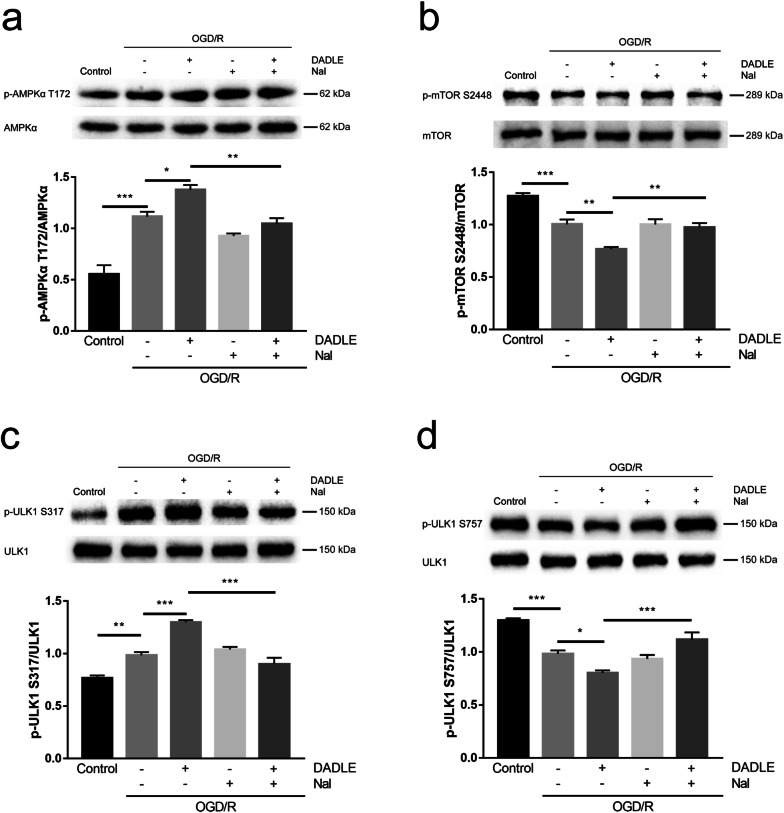
DOR activation induced protective AMPK/mTOR/ULK1 signaling to enhance autophagy after OGD/R injury. Western blot analysis of p-AMPKα T172 (**a**), p-mTOR S2448 (**b**), p-ULK1 S317 (**c**) and p-ULK1 S757 (**d**) levels in SH-SY5Y cells (N = 6 per group). The values are shown as the mean ± SEM. Significant differences: the OGD/R group vs. the DADLE group (*p < 0.05) and the naltrindole group (p > 0.05, ns), the DADLE group vs. the DADLE + naltrindole group (**p < 0.01) in **a**; the OGD/R group vs. the DADLE group (**p < 0.01) and the naltrindole group (p > 0.05, ns), the DADLE group vs. the DADLE + naltrindole group (**p < 0.01) in **b**; the OGD/R group vs. the DADLE group (***p < 0.001) and the naltrindole group (p > 0.05, ns), the DADLE group vs. the DADLE + naltrindole group (***p < 0.001) in **c**; the OGD/R group vs. the DADLE group (*p < 0.05) and the naltrindole group (p > 0.05, ns), the DADLE group vs. the DADLE + naltrindole group (***p < 0.001) in **d**. *Nal* naltrindole

We further assessed the changes in the AMPK/mTOR/ULK1 signaling pathway following OGD/R injury and DADLE treatment in vitro. Compared with those in the control group, the phosphorylation levels of AMPKα T172 in the OGD/R group and the naltrindole group were significantly increased (Fig. 7a). Consistent with the in vivo results, it was found that the p-AMPKα T172 level was further increased in the DADLE group compared to the OGD/R group and that this increase was reversed in the DADLE + naltrindole group (Fig. 7a). The phosphorylation levels of mTOR S2448 in the OGD/R group, naltrindole group and DADLE + naltrindole group were markedly decreased compared to those in the control group, while there was a further decrease in the p-mTOR S2448 level in the DADLE group (Fig. 7b). The level of p-ULK1 S317 was obviously increased in the OGD/R group compared with the control group and was further elevated in the DADLE group, while the p-ULK1 S317 levels were lower in the naltrindole group and DADLE + naltrindole group than in the DADLE group (Fig. 7c). In contrast, the p-ULK1 S757 levels in the OGD/R group and naltrindole group were lower than those in the control group, decreased further in the DADLE group and increased again in the DADLE + naltrindole group (Fig. 7d). These results indicate that OGD/R injury induced autophagy via the AMPK/mTOR/ULK1 signaling pathway, possibly as an adaptive endogenous reaction against OGD/R stress, whereas DOR activation augmented autophagic activity via modulation of the AMPK/mTOR/ULK1 axis and exerted neuroprotective effects against OGD/R injury.

**Fig. 8:**
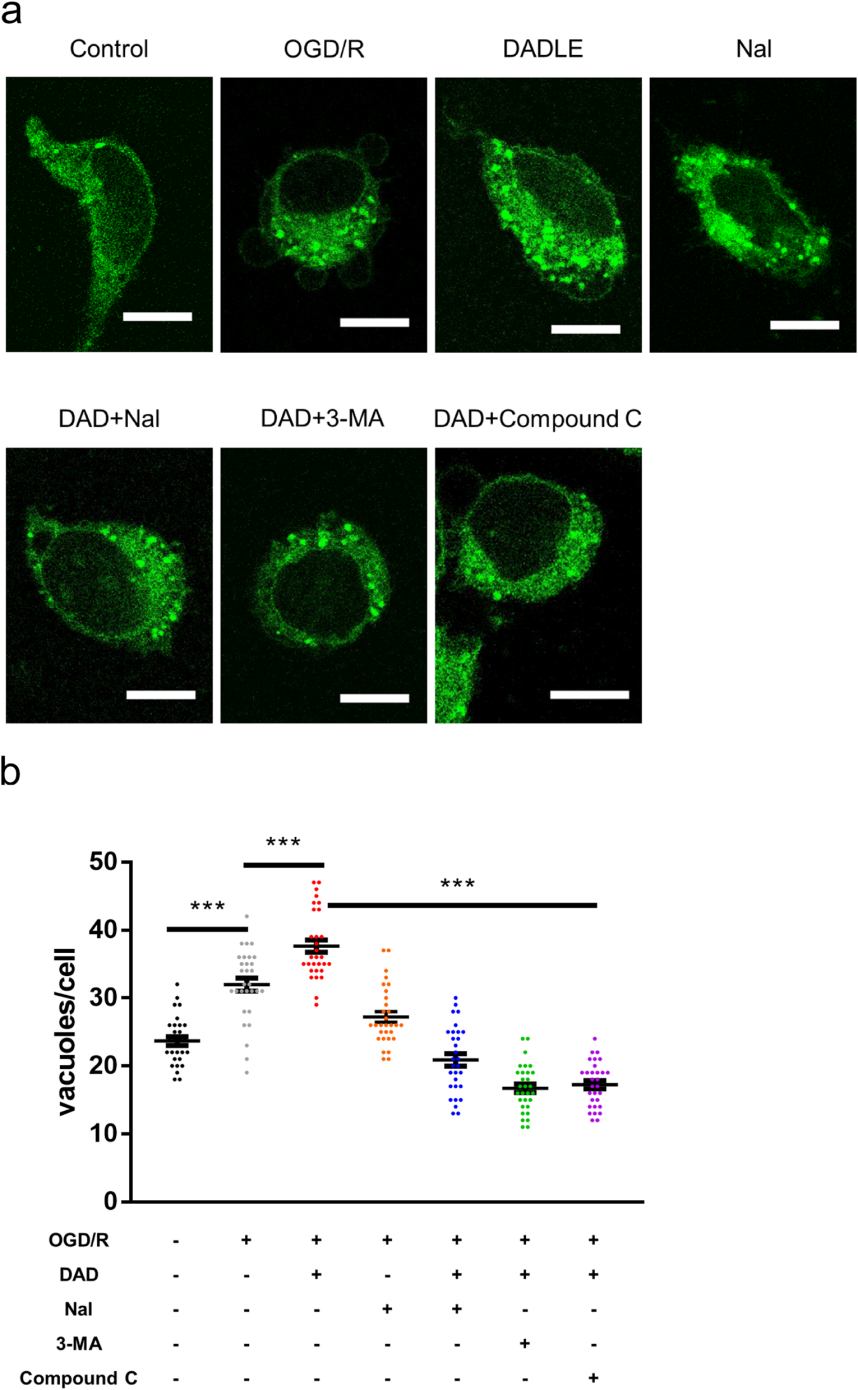
The effects of AMPK on DOR-mediated autophagy in the OGD/R model. **a** Images of MDC staining of SH-SY5Y cells exposed to different treatments. **b** Quantitative analysis of MDC-positive vacuoles in cells (N = 30 per group). Scale bar = 10 μm. The values are shown as the mean ± SEM. Significant differences: the OGD/R group vs. the DADLE group and the naltrindole group (***p < 0.001), the DADLE group vs. the DADLE + naltrindole group, the DADLE + 3-MA group and the DADLE + compound C group (***p < 0.001) in **b**. *DAD* DADLE, *Nal* naltrindole

The MDC staining assay was subsequently performed to examine whether AMPK is the major player in the AMPK/mTOR/ULK1 signaling axis responsible for DADLE-mediated autophagy. As shown in Fig. 8a, normal SH-SY5Y cells exhibited distinct branches with a few bright green dots in the cytoplasm, while OGD/R-exposed cells shrank into spheres with many green dots in the cytoplasm, indicating that autophagy was activated by OGD/R. Numerous bright green dots appeared in the cytoplasm of the cells following DADLE treatment (Fig. 8a, b). In contrast, OGD/R-exposed cells in the naltrindole group, DADLE + naltrindole group, DADLE + 3-MA group and DADLE + compound C group displayed a few obvious green dots in the cytoplasm (Fig. 8a, b). These results suggest that AMPK plays a crucial role in the AMPK/mTOR/ULK1 signaling axis during DOR-evoked autophagy after OGD/R injury.

## Discussion

Our previous study showed that DOR activation by DADLE improves ischemic neuronal survival [7], which was in accordance with the results shown in Fig. 3c. In vitro results in SH-SY5Y cells also revealed that DADLE treatment obviously increased the viability of OGD/R cells (Fig. 2b). However, the beneficial effects of DADLE were abolished by the DOR inhibitor naltrindole (Figs. 2c, 3c), indicating that DADLE-induced DOR activation exerts a neuroprotective effect against ischemia. DOR is an oxygen-sensitive protein, and the mRNA and protein levels of DOR in neurons are decreased by hypoxia [9, 12]. Similar to hypoxic injury, cerebral ischemia caused by asphyxial cardiac arrest reduces DOR expression in the rat hippocampus [23]. In vivo and vitro studies showed that the DOR level was significantly downregulated under ischemic stress and was obviously rescued following DADLE treatment (Fig. 2d, e), suggesting that the agonistic effects of DADLE partially restored the impaired DOR level induced by ischemia. Although the specific mechanisms were unclear, there was evidence that internalized DOR induced by a delta opioid peptide was recycled to the cell membrane for a new signaling cycle via an intracellular sorting mechanism [26] and that DOR activation triggered intracellular prosurvival signaling pathways (such as the ERK cascade) to promote ischemic neuronal survival [10, 12], which might be implicated in DADLE-modulated upregulation of DOR. Notably, there was a significant decrease in the DOR level following the addition of naltrindole (Fig. 2d, e), which is inconsistent with previous reports showing that the DOR level is unchanged by a combination of DADLE and naltrindole [23]. This discrepancy could be explained by differences in the time points of observation. Neuronal loss is observed in the hippocampal CA1 region from day 3 after ischemia [27]; however, Gao et al. observed changes in DOR levels only within 24 h postischemia, which is different from our observation. Furthermore, 50 nmol DADLE was used by Gao et al., while our previous study revealed that high doses of DADLE (25 nmol) aggravate cerebral ischemic injury after 3 days [7]; thus, the dose of DADLE may be another factor that influenced the results.

Growing evidence has revealed that autophagy is closely related to ischemic injury and occurs in both ischemic neurons and astrocytes [18, 19]. Autophagic activation is involved in the neuroprotective effects of nicotinamide phosphoribosyltransferase and hamartin against cerebral ischemia [15, 28]. In addition, the upregulation of autophagic flux in astrocytes protects neurons from I/R injury [17]. In a study on the cardioprotective effects of DOR, DADLE treatment was shown to enhance autophagy to alleviate lipopolysaccharide (LPS)-induced myocardial injury [29]. Our previous findings revealed that DADLE-evoked DOR activation exerts a cytoprotective effect by inducing autophagy in OGD-exposed astrocytes [11]. Western blotting results showed that the LC3 II/I level was significantly elevated in the I/R group compared with the sham group, while P62 was significantly reduced (Fig. 3a, b), indicating that endogenous autophagy was induced under ischemic stress. The changes in LC3 and P62 in the DADLE group were more pronounced than those in the I/R group and were reversed in the DADLE + naltrindole group (Fig. 3a, b), suggesting that postischemic autophagy was enhanced by DOR activation. Furthermore, the inhibition of autophagy via the addition of 3-MA abolished the neuronal protection mediated by DADLE in the ischemic hippocampus (Fig. 3c, d), implying that DOR-induced autophagy contributed to the improvement in ischemic neuronal survival. Moreover, DOR-mediated autophagy contributed to the protective actions of myocardial and cerebral ischemia, showing that autophagy may play a universal role in the anti-ischemic mechanisms of DOR; however, more evidence is needed to confirm this speculation.

Similar effects of autophagic activation were observed in the OGD/R model as well (Fig. 4a, b). Notably, the combination of DADLE and naltrindole did not reduce the LC3 II/I level, which is inconsistent with the change in P62 (Fig. 4a, b). LC3 II, which is formed by the conjugation of LC3-I to phosphatidylethanolamine, is required for the elongation of phagophores and maturation of autophagosomes, while the adaptor protein P62 delivers an intracellular component to the autophagosome and is eventually degraded in autolysosomes [30]. Since LC3 and P62 play different roles in the autophagic process, high LC3 II/I and P62 levels indicate abnormal autophagic flux. Autophagic flux dysfunction is observed in hippocampal neurons after OGD/R injury [31]. Autophagic activation was obvious in SH-SY5Y cells infected with mCherry-GFP-LC3B from each experimental group following OGD/R treatment, as evidenced by many fluorescent dots, but the percentage of red and yellow dots in the cells was different among the experimental groups (Fig. 4c, d). The percentage of red dots in the DADLE group was far exceeded that in the OGD/R group and DADLE + naltrindole group (Fig. 4d). The above results indicate that OGD/R injury caused autophagic flux impairment, while DOR activation ameliorated autophagic flux dysfunction and promoted autophagic degradation. The occurrence of autophagic flux dysfunction in cells but not animals may reflect the differences between the cell model and the animal model. The response of SH-SY5Y cells to ischemic stress was more direct than that of in neurons in vivo, which exhibited more moderate changes, which may have been restricted by homeostasis regulation. Together, the in vivo and in vitro results provide valuable evidence that although autophagic activation induced by I/R injury is insufficient to protect against neuronal death, DOR activation with DADLE further enhance neuronal autophagy and alleviate autophagic flux dysfunction, eventually alleviating I/R injury and promoting neuronal survival.

There was no obvious neuronal damage to the CA3 region or DG (Additional files 1, 2: Figs. S1, S2), indicating that CA3 and DG neurons are more resistant to ischemia than CA1 neurons. Previous work has strongly suggested that although the hippocampus is vulnerable to ischemia, the ischemic tolerance of the CA1, CA3 and DG subregions are different [32]. CA3 and DG neurons are more resistant to ischemia than CA1 neurons [32, 33]. Furthermore, hamartin acts through mTORC1-dependent autophagy to exert anti-ischemic effects in CA3 neurons, while similar neuroprotection in CA1 neurons is triggered by ischemic preconditioning [28]. The unique mechanisms underlying the ischemic tolerance of the CA3 and DG subregions are worth further investigation.

DOR expression in the brains of hypoxia-tolerant turtles is much higher than that in the brains of rats [34], while DADLE acts as a hibernation trigger; DOR activation by DADLE reduces the metabolism of hibernating animals and induces hibernation [35], showing the important role of DOR in energy metabolism. In addition, AMPK, as a “cell energy sensor”, is key for the regulation of cell energy metabolism [20]. An in vitro study found that DOR activation increases AMPKα phosphorylation at T172 and enhances AMPK activity through synergistic signaling with a Gq/11-coupled receptor [36]. Furthermore, AMPK signaling plays a vital role in regulating autophagy [20] and exerts neuroprotective effects against ischemic stroke [37]. AMPK and mTOR regulate autophagy through phosphorylation of ULK1 [25]. AMPK directly phosphorylates ULK1 at S317, inducing downstream autophagy and also inhibits mTOR activity and indirectly reduces mTOR-evoked phosphorylation of ULK1 at S757, thereby enhancing the interaction between AMPK and ULK1 [25]. The protein activities of AMPK and mTOR are regulated by phosphorylation of AMPKα T172 and mTOR S2448, respectively [38, 39].

AMPK signaling is involved in ischemia-induced autophagy activation [16]. The phosphorylation level of AMPKα T172 in the CA1 region increases rapidly within 6 h after global ischemia and falls to normal level at 24 h, while the p-ULK1 S317 level significantly is increased from 3 h to 24 h; concomitantly, the levels of mTOR and p-ULK1 S757 decrease progressively within 24 h [16]. The early increase in AMPK signaling promotes postischemic autophagy and exerts neuroprotective effects, while autophagic activation is accompanied by inactivation of AMPK signaling 24 h after ischemia, suggesting that the decrease in p-AMPK precedes or coincides with increases in autophagy markers [16]. Inconsistent with the changes that occur within 24 h after ischemia, our results revealed that the phosphorylation level of AMPKα T172 in the hippocampus was significantly decreased on day 3 postischemia, there was no change in the p-mTOR S2448 level, and phosphorylation of ULK1 S317 and ULK1 S757 was obviously increased (Fig. 5). The decreased p-AMPKα T172 level and elevated level of p-ULK1 S317 implies that the modulatory effects of AMPK signaling were exhausted, while ischemia-induced autophagy continued until the 3rd day after ischemia. Moreover, the level of p-mTOR S2448 was close to the normal level, and the p-ULK1 S757 level was significantly increased, implying that mTOR signaling began to inhibit autophagy, further revealing that the modulatory effect of AMPK signaling on autophagic activation was weakened. In the DADLE group compared to the I/R group, the phosphorylation level of AMPKα T172 was obviously increased, the p-ULK1 S317 level was further increased, and the levels of p-mTOR S2448 and p-ULK1 S757 were significantly decreased; however, these changes were reversed by naltrindole (Fig. 5), showing that DOR activation augmented autophagic activity by triggering the AMPK/mTOR/ULK1 signaling pathway. Furthermore, the combination of DADLE and compound C abolished the neuroprotective effects of DOR activation on ischemic CA1 neurons (Fig. 6), reflecting the important role of AMPK in DADLE-mediated neuroprotection against ischemia.

The in vitro results revealed that OGD/R treatment elevated the phosphorylation levels of AMPKα T172 and ULK1 S317 and reduced the levels of p-mTOR S2448 and p-ULK1 S757 (Fig. 7). Although this result is not completely consistent with the in vivo results (Fig. 5), OGD/R-induced autophagy was also triggered by the AMPK/mTOR/ULK1 signaling pathway, reflecting the intrinsic connection between the cell model and animal model. The phosphorylation levels of AMPKα T172 and ULK1 S317 in the DADLE group were further increased compared with those in the OGD/R group, and the levels of p-mTOR S2448 and p-ULK1 S757 were further decreased; however, these changes were reversed by the combination of DADLE and naltrindole (Fig. 7), indicating that DOR activation further augmented autophagy through the AMPK/mTOR/ULK1 signaling pathway. Furthermore, the combination of DADLE and compound C decreased the DOR-induced autophagic enhancement (Fig. 8), reflecting the important role of AMPK in DOR-mediated autophagy. The in vivo and vitro results thus reveal that DOR activation by DADLE enhances neuronal autophagy to protect against cerebral ischemia by activating the AMPK/mTOR/ULK1 signaling pathway.

In summary, the present study showed that the agonistic effects of DADLE can partially restore the reduced DOR levels in vivo in the ischemic hippocampus and in vitro in an OGD/R cell model. Furthermore, it was found that DOR-mediated autophagic enhancement participates in neuronal protection against I/R injury in vivo and vitro and that the AMPK/mTOR/ULK1 signaling pathway plays a vital role in DOR-evoked autophagy regulation.

## Conclusions

**Fig. 9:**
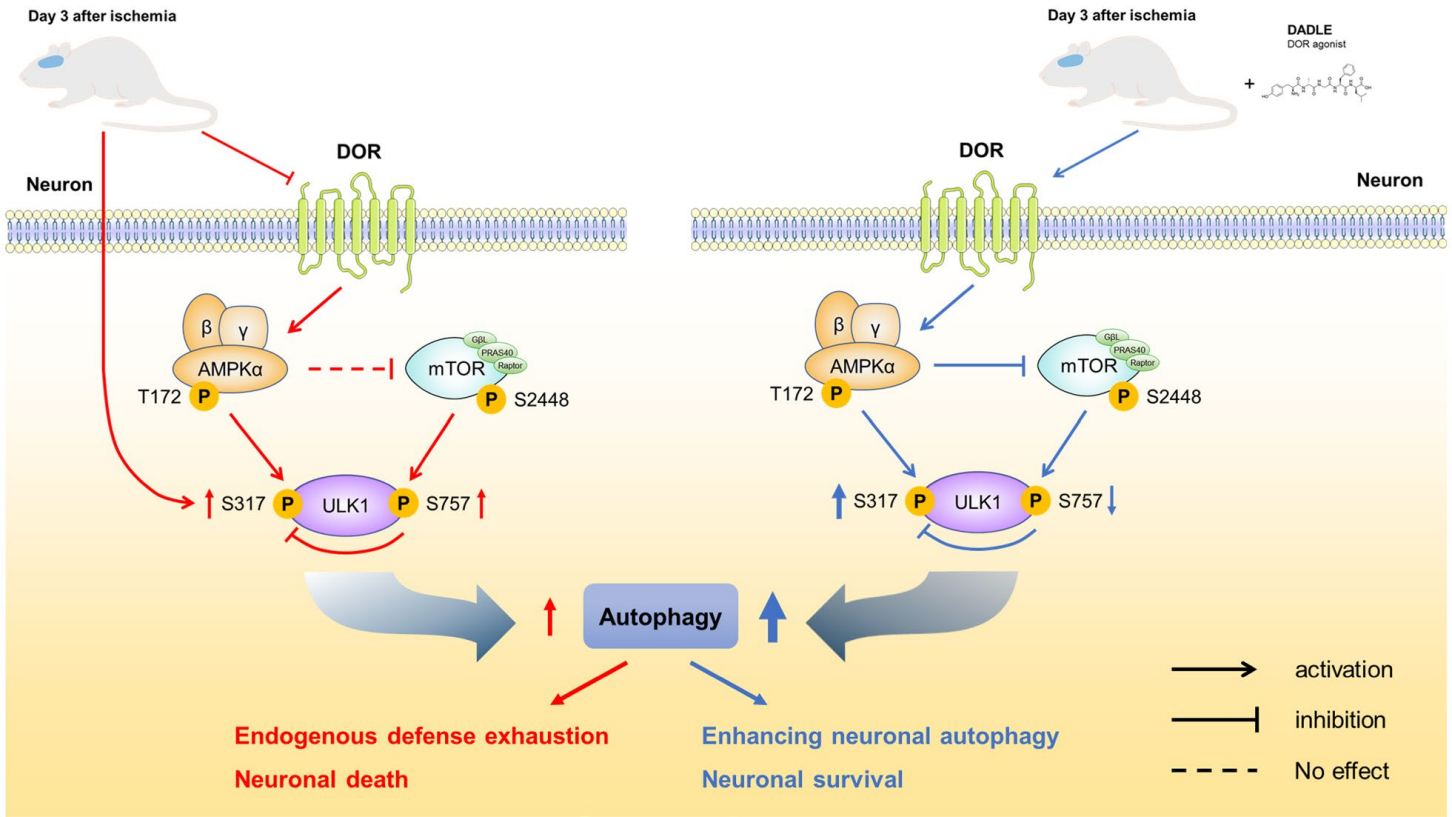
Diagram of the proposed mechanistic signaling pathway. Although cerebral ischemia leads to autophagic activation, it reduces the DOR level and inhibits AMPK activity, leading to endogenous defense exhaustion and eventually neuronal death (left). However, DOR activation by DADLE triggers AMPK/mTOR/ULK1 signaling, further enhancing neuronal autophagy and alleviating autophagic flux dysfunction to ameliorate ischemic neuronal survival (right)

As illustrated in Fig. 9, our results demonstrate that DOR activation by DADLE augments neuronal autophagic activity in an AMPK/mTOR/ULK1 signaling pathway-dependent manner to promote ischemic CA1 neuronal survival. These results provide new evidence for DADLE-mediated neuroprotection against ischemia, revealing the effects of the DOR-AMPK-autophagy axis on ischemic neuronal survival and further expanding our understanding of the anti-ischemic mechanisms of DOR.

## Supplementary information

**Additional file 1: Fig. S1.** The effects of autophagy on neuronal survival in the hippocampal CA3 region on day 3 postischemia. (a) Immunofluorescence images of NeuN-positive neurons. There was no significant difference in neuronal morphology between the different groups. Scale bar = 100 μm. (b) Quantitative analysis of neuron counts (N = 3 per group). DAD: DADLE; Nal: Naltrindole.

**Additional file 2: Fig. S2.** The effects of autophagy on neuronal survival in the hippocampal DG region on day 3 postischemia. (a) Immunofluorescence images of NeuN-positive neurons. There was no significant difference in neuronal morphology between the experimental groups. Scale bar = 100 μm. (b) Quantitative analysis of neuron counts (N = 3 per group). DAD: DADLE; Nal: Naltrindole.

## Data Availability

The datasets used and analyzed in the current study are available from the corresponding author in response to reasonable requests.

## Abbreviations

CCK-8: Cell Counting Kit-8
DADLE: [d-Ala2, d-Leu5] enkephalin
DG: Dentate gyrus
DOR: Delta opioid receptor
GPCR: G protein-coupled receptor
I/R: Ischemia/reperfusion
LPS: Lipopolysaccharide
MCAO: Middle cerebral artery occlusion
MDC: Monodansylcadaverine
MOI: Multiplicity of infection
NeuN: Neuron-specific nuclei protein
OD: Optical density
OGD: Oxygen–glucose deprivation
OGD/R: Oxygen–glucose deprivation/reperfusion
PBS: Phosphate buffer saline
PFA: Paraformaldehyde
3-MA: 3-Methyladenine
4-VO: Four-vessel occlusion
