# Supplementary figures and images for: Delta opioid peptide [d-Ala2, d-Leu5] enkephalin confers neuroprotection by activating delta opioid receptor-AMPK-autophagy axis against global ischemia
Source: Cell Biosci. 2020 Jun 15;10:79. doi: 10.1186/s13578-020-00441-z (PMC7294676; doi:10.1186/s13578-020-00441-z)

a

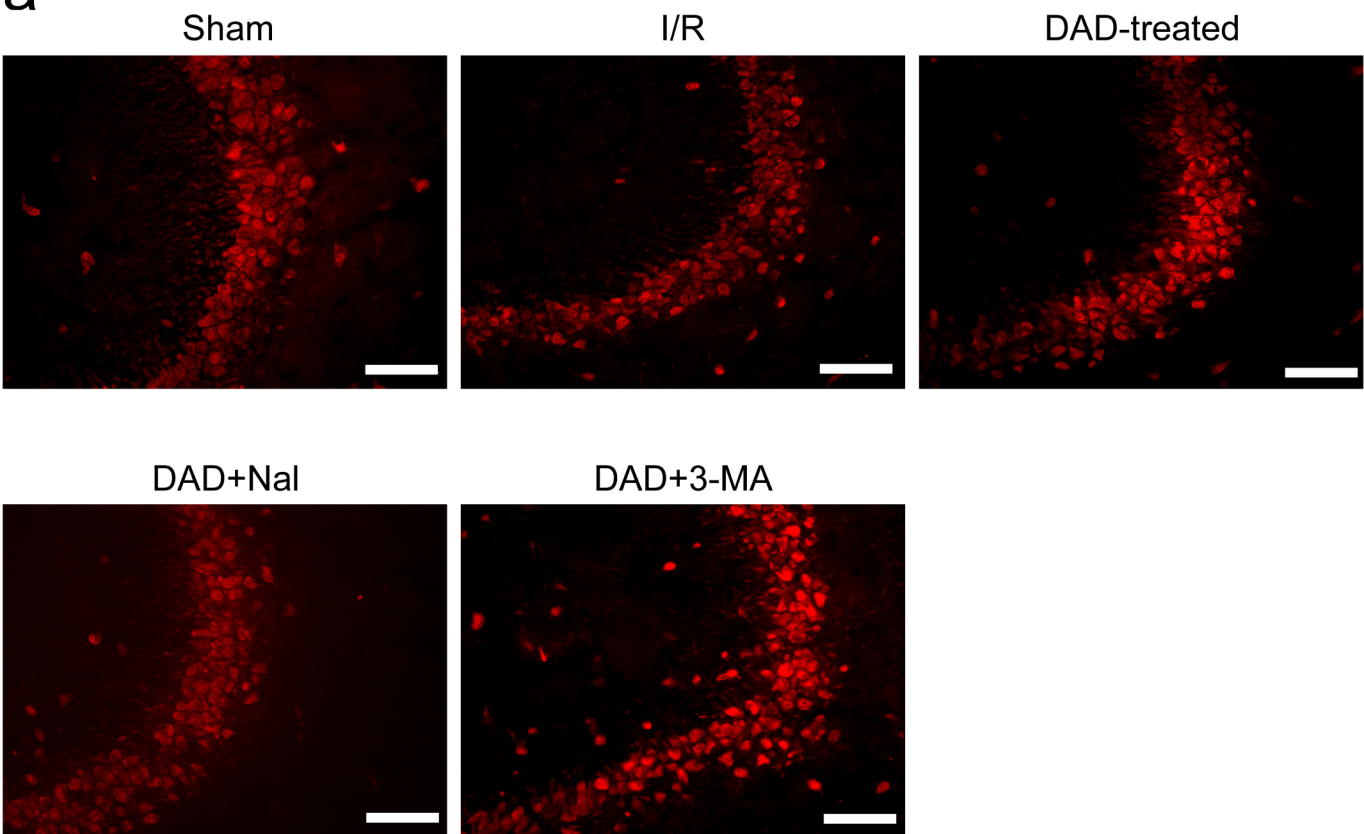

b

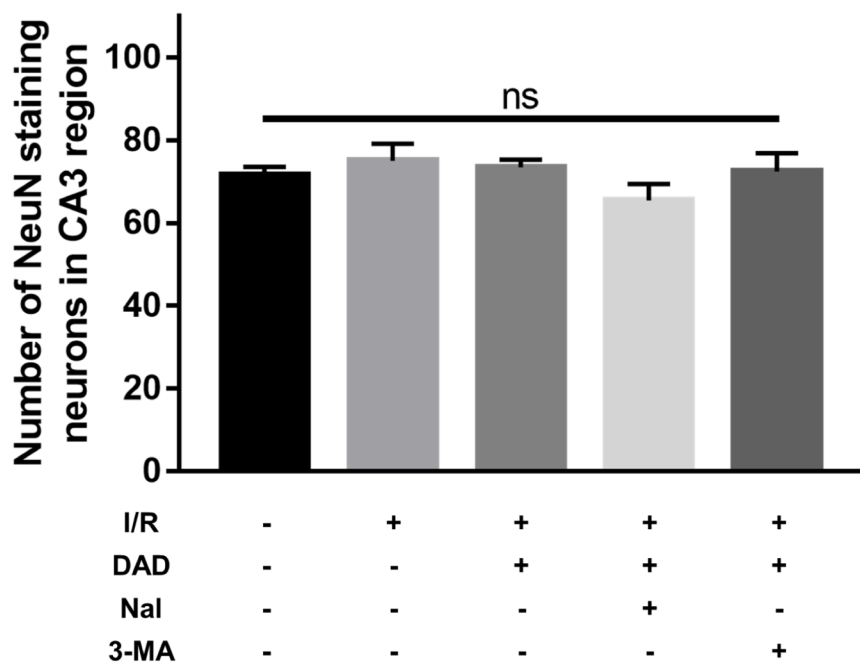

Supplement: Supplementary file 1 — Additional file 1: Fig. S1. The effects of autophagy on neuronal survival in the hippocampal CA3 region on day 3 postischemia. (a) Immunofluorescence images of NeuN-positive neurons. There was no significant difference in neuronal morphology between the different groups. Scale bar = 100 μm. (b) Quantitative analysis of neuron counts (N = 3 per group). DAD: DADLE; Nal: Naltrindole. [file 13578_2020_441_MOESM1_ESM.pdf]

a

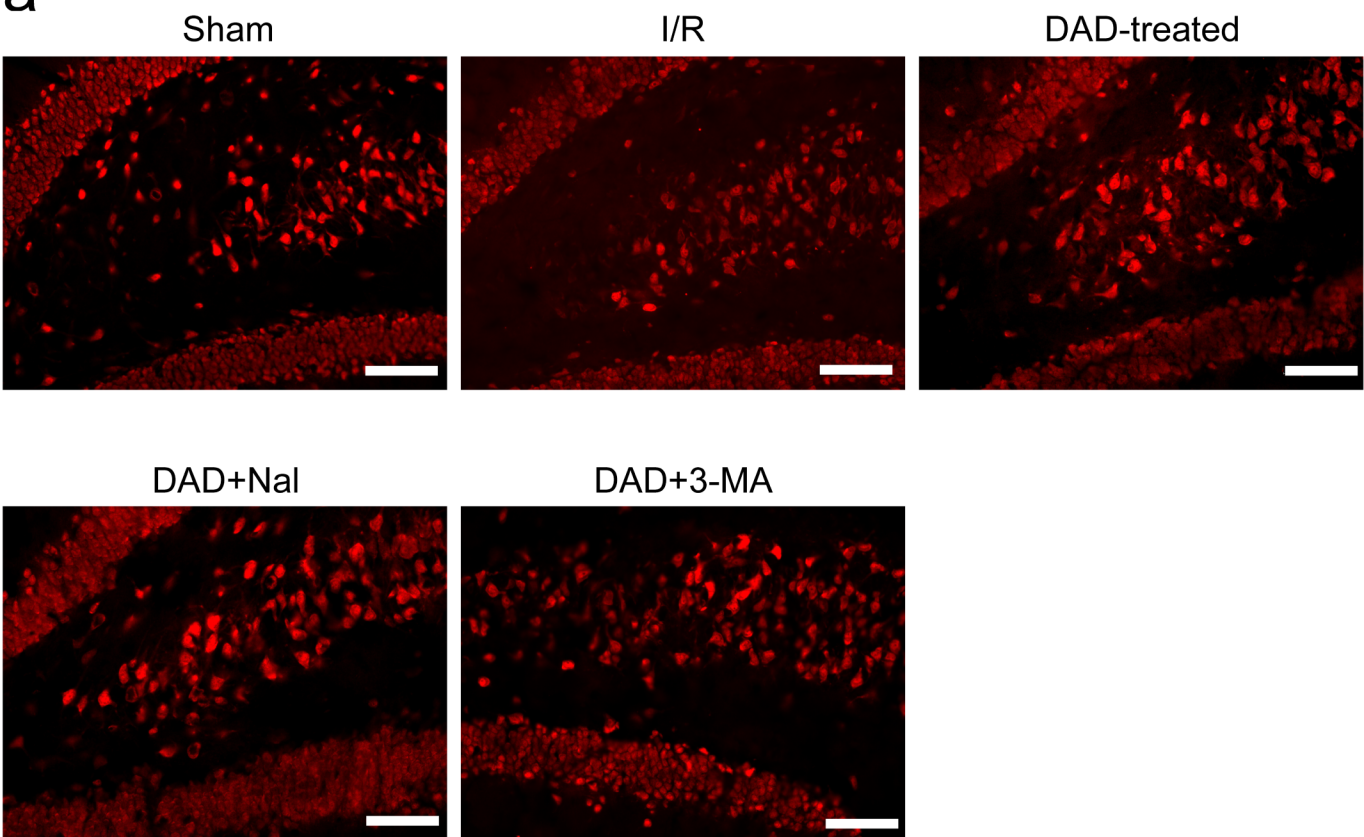

b

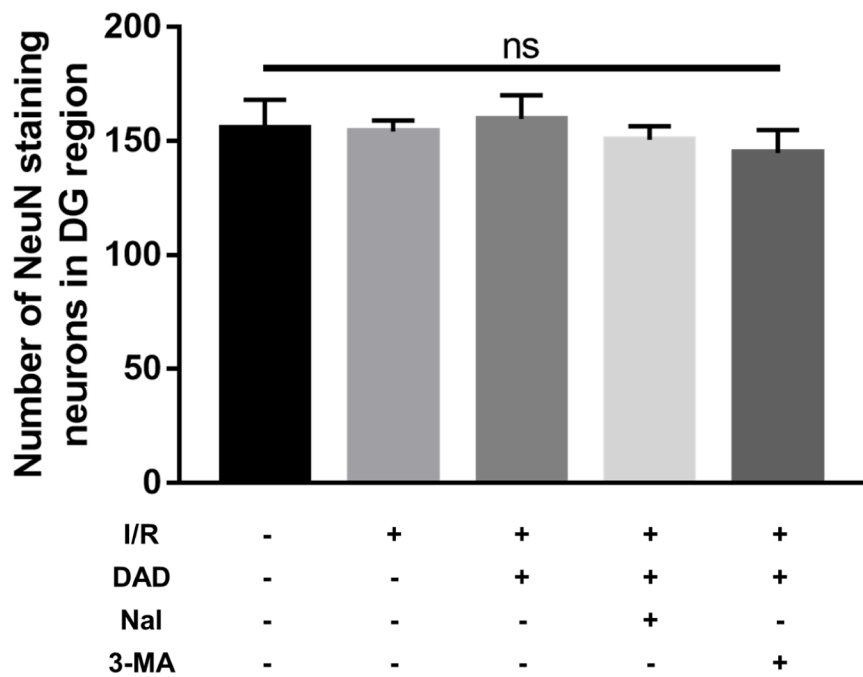

Supplement: Supplementary file 2 — Additional file 2: Fig. S2. The effects of autophagy on neuronal survival in the hippocampal DG region on day 3 postischemia. (a) Immunofluorescence images of NeuN-positive neurons. There was no significant difference in neuronal morphology between the experimental groups. Scale bar = 100 μm. (b) Quantitative analysis of neuron counts (N = 3 per group). DAD: DADLE; Nal: Naltrindole. [file 13578_2020_441_MOESM2_ESM.pdf]
